# Normal glucose tolerant women with low glycemia during the oral glucose tolerance test have a higher risk to deliver a low birth weight infant

**DOI:** 10.3389/fendo.2023.1186339

**Published:** 2023-06-02

**Authors:** Lore Raets, Lore Van Doninck, Paul Van Crombrugge, Carolien Moyson, Johan Verhaeghe, Sofie Vandeginste, Hilde Verlaenen, Chris Vercammen, Toon Maes, Els Dufraimont, Nele Roggen, Christophe De Block, Yves Jacquemyn, Farah Mekahli, Katrien De Clippel, Annick Van Den Bruel, Anne Loccufier, Annouschka Laenen, Roland Devlieger, Chantal Mathieu, Katrien Benhalima

**Affiliations:** ^1^ Department of Endocrinology, University Hospital Gasthuisberg, KU Leuven, Leuven, Belgium; ^2^ Medicine, KU Leuven, Leuven, Belgium; ^3^ Department of Endocrinology, Onze-Lieve-Vrouwziekenhuis (OLV) Ziekenhuis Aalst-Asse-Ninove, Aalst, Belgium; ^4^ Department of Obstetrics & Gynecology, Universitair Ziekenhuis (UZ) Gasthuisberg, KU Leuven, Leuven, Belgium; ^5^ Department of Obstetrics & Gynecology, Onze-Lieve-Vrouwziekenhuis (OLV) Ziekenhuis Aalst-Asse-Ninove, Aalst, Belgium; ^6^ Department of Endocrinology, Imelda Ziekenhuis, Bonheiden, Belgium; ^7^ Department of Obstetrics & Gynecology, Imelda Ziekenhuis, Bonheiden, Belgium; ^8^ Department of Endocrinology-Diabetology-Metabolism, Antwerp University Hospital, Edegem, Belgium; ^9^ Department of Obstetrics & Gynecology, Antwerp University Hospital, Edegem, Belgium; ^10^ Antwerp Surgical Training, Anatomy and Research Centre (ASTARC) and Global Health Institute (GHI), Antwerp University University of Antwerp (UA), Antwerp, Belgium; ^11^ Department of Endocrinology, Kliniek St-Jan Brussel, Brussel, Belgium; ^12^ Department of Obstetrics & Gynecology, Kliniek St-Jan Brussel, Brussel, Belgium; ^13^ Department of Endocrinology, Algemeen Ziekenhuis (AZ) St. Jan Brugge, Brugge, Belgium; ^14^ Department of Obstetrics & Gynecology, Algemeen Ziekenhuis (AZ) St. Jan Brugge, Brugge, Belgium; ^15^ Center of Biostatics and Statistical bioinformatics, KU Leuven, Leuven, Belgium

**Keywords:** low glycemia, normal glucose tolerant, pregnancy outcomes, oral glucose tolerance test, low birth weight

## Abstract

**Background:**

Data are limited on pregnancy outcomes of normal glucose tolerant (NGT) women with a low glycemic value measured during the 75g oral glucose tolerance test (OGTT). Our aim was to evaluate maternal characteristics and pregnancy outcomes of NGT women with low glycemia measured at fasting, 1-hour or 2-hour OGTT.

**Methods:**

The Belgian Diabetes in Pregnancy-N study was a multicentric prospective cohort study with 1841 pregnant women receiving an OGTT to screen for gestational diabetes (GDM). We compared the characteristics and pregnancy outcomes in NGT women according to different groups [(<3.9mmol/L), (3.9-4.2mmol/L), (4.25-4.4mmol/L) and (>4.4mmol/L)] of lowest glycemia measured during the OGTT. Pregnancy outcomes were adjusted for confounding factors such as body mass index (BMI) and gestational weight gain.

**Results:**

Of all NGT women, 10.7% (172) had low glycemia (<3.9 mmol/L) during the OGTT. Women in the lowest glycemic group (<3.9mmol/L) during the OGTT had compared to women in highest glycemic group (>4.4mmol/L, 29.9%, n=482), a better metabolic profile with a lower BMI, less insulin resistance and better beta-cell function. However, women in the lowest glycemic group had more often inadequate gestational weight gain [51.1% (67) vs. 29.5% (123); p<0.001]. Compared to the highest glycemia group, women in the lowest group had more often a birth weight <2.5Kg [adjusted OR 3.41, 95% CI (1.17-9.92); p=0.025].

**Conclusion:**

Women with a glycemic value <3.9 mmol/L during the OGTT have a higher risk for a neonate with birth weight < 2.5Kg, which remained significant after adjustment for BMI and gestational weight gain.

## 1 Introduction

In contrast to women with gestational diabetes (GDM) who have higher glucose levels and an increased risk for large-for-gestational age (LGA) neonates (1, 2), women with low glycemia may be at increased risk to deliver neonates with a low birth weight. It has been demonstrated that infants born with a birth weight <2.5Kg are at increased risk to develop an adverse metabolic profile later in life with increased risk to develop type 2 diabetes (T2DM) and cardiovascular disease (3–6). Low birth weight can be caused by maternal conditions such as placental dysfunction, malnutrition or impaired maternal metabolism (7, 8). Glucose diffuses from the mother to the fetus by placental transport mediated by glucose transporters (GLUT)-1, GLUT-4 and GLUT-9 {Stanirowski, (9) #14}. Since the fetus’ blood glucose level is proportional to the blood glucose level of the mother, hypoglycemia might increase the risk for various adverse pregnancy outcomes such as low-birth-weight or small-for-gestational age (SGA) neonates (7, 8, 10, 11).

However, only few studies have focused on the potential relationship between low glycemia during the oral glucose tolerance test (OGTT) and the impact on maternal and neonatal outcomes in normal glucose tolerant (NGT) women. Studies focused mostly on the effects of reactive hypoglycemia during an OGTT (7, 8, 12–16) and data are limited on the potential effects of lower glycemic values in general. In addition, these studies reported conflicting results concerning the impact on maternal and neonatal outcomes, especially on neonatal birth weight (7, 12–14). Moreover, most studies investigated the effects of hypoglycemia in women with GDM or obesity (12, 14, 16, 17). Data are sparse on pregnancy outcomes of NGT women with a lower glycemic value measured during the OGTT between 24-28 weeks of pregnancy.

We aimed therefore to evaluate maternal characteristics and pregnancy outcomes in a large cohort of NGT women with a low glycemic value, being less than the American Diabetes Association (ADA) cut-off for hypoglycemia outside pregnancy (<3.9mmol/L), measured at any stage (fasting, 1-hour or 2-hour) during the 75g OGTT used for screening for GDM during pregnancy (18). In addition, we also aimed to evaluate maternal characteristics and pregnancy outcomes across different groups of low glycemia [stratified according to quartiles of glycemic value (<3.9mmol/L), (3.9-4.2mmol/L), (4.3-4.4mmol/L) and (>4.4mmol/L)] during the OGTT.

## 2 Subjects and methods

### 2.1 Study design and setting

This is a sub-analysis of the Belgian Diabetes in Pregnancy-North (BEDIP-N) study. The BEDIP-N study was a multicentric prospective cohort study to evaluate different screening strategies for GDM that has previously been described in detail (19–25). The BEDIP-N study was approved by the Institutional Review Boards of all participating centers and all investigations have been carried out in accordance with the principles of the Declaration of Helsinki as revised in 2008. Before inclusion to the study, informed consent was obtained. Participants were enrolled between 6 and 14 weeks of pregnancy, when fasting plasma glucose (FPG) was measured. Women with impaired fasting glycemia or diabetes in early pregnancy according to the ADA criteria were excluded (26). Women without (pre)diabetes received universal screening for GDM between 24-28 weeks of pregnancy with a non-fasting 50g glucose challenge test (GCT) followed by a 75g 2-hour OGTT. Results of the GCT were blinded for participants and health care providers. All participants received an OGTT irrespective of the GCT result. Glucose was measured in fluoride-oxalate tubes, limiting the risk for false low glucose values as fluoride inhibits glycolysis. The OGTT was performed according to standard operating procedures provided to each participating center and blood samples were immediately sent to the laboratory for analyzes. The 2013 World Health Organization (WHO) criteria were used for the diagnosis of GDM (19, 20, 27). The ADA-recommended glycemic targets were used for the treatment of GDM (27).

According to the ADA, hypoglycemia in pregnancy is defined as a value <3.5 mmol/L (63 mg/dl), whereas a value <3.9 mmol/L (70 mg/dl) is considered as a low glycemic value or level one hypoglycemia in pregnancy (18, 28, 29), while a value < 3.0 mmol/L (54 mg/dl) is classified as a level two hypoglycemia (18, 28, 29). In addition, we also divided the cohort into groups of low glycemia [stratified according to quartiles of the glycemic value (<3.9mmol/L), (3.9-4.2mmol/L), (4.3-4.4mmol/L) and (>4.4mmol/L)] during the OGTT to evaluate maternal characteristics and pregnancy outcomes across these different groups.

In total, 1841 women received an OGTT, of which 12.4% (n=229) were diagnosed with GDM and 1612 women were NGT. Because only four women in the GDM-group had low glycemia (<3.9 mmol/L) at fasting, 1-hour or 2-hour OGTT, we excluded the GDM-group for further analysis.

### 2.2 Study visits and measurements

Baseline characteristics and obstetrical history were collected at first visit (19). Anthropometric measurements were obtained, and several self-administered questionnaires were completed at first visit (6-14 weeks of pregnancy) and at the time of the OGTT (26-28 weeks of pregnancy) (19).

Blood pressure (BP) was measured using an automatic BP monitor. A BMI ≥ 25 kg/m² was defined as overweight and a BMI ≥ 30 kg/m² was defined as obesity based on the measured BMI at first prenatal visit. During this visit, a fasting blood test was taken to measure FPG, insulin, lipid profile [total cholesterol, high density lipoproteins (HDL), low density lipoproteins (LDL) cholesterol and triglycerides], and hemoglobin A1c (HbA1c). The homeostasis model assessment of insulin resistance (HOMA-IR) and beta-cell function (HOMA-B) were measured in early pregnancy (30). At the visit with OGTT in pregnancy, a fasting lipid profile, HbA1c and different indices of beta-cell function [HOMA-B, the insulinogenic index divided by HOMA-IR and the insulin secretion-sensitivity index-2 (ISSI-2)] were measured (30, 31). In addition, indices of insulin sensitivity were measured, such as the Matsuda index, a measure of whole body insulin sensitivity, and the reciprocal of the HOMA-IR index (19, 30–34).

At first visit and at the time of the OGTT, a food questionnaire was used to question servings per week of different important food categories and beverages (35). Less healthy consumption was assigned 0 or -1 points. By summing up the points for all 14 food groups, the diet score could range from -12 to 15. At the time of the OGTT, the International Physical Activity Questionnaire (IPAQ) questionnaire (validated for the Belgian population) assessed physical activity (19, 36). Results of the IPAQ were reported in categories (low, moderate or high activity levels) as previously reported (37).

### 2.3 Pregnancy and delivery outcome data

Following pregnancy outcomes were collected: gestational age, preeclampsia (*de novo* BP ≥140/90mmHg > 20 weeks with proteinuria or signs of end-organ dysfunction), gestational hypertension (*de novo* BP ≥140/90mmHg > 20 weeks), type of labor and type of delivery, macrosomia (>4 kg), LGA defined as birth weight >90 percentile according to standardized Flemish birth charts adjusted for sex of the baby and parity (38), SGA defined as birth weight <10 percentile according to standardized Flemish birth charts adjusted for sex of the baby and parity (38), low birth weight defined as a birth weight <2.5kg, preterm delivery (<37 completed weeks), shoulder dystocia and admission on the neonatal intensive care unit (NICU) (19). A glycemic value <2.2 mmol/L was considered as a neonatal hypoglycemia across all centers, irrespective of the need for intravenous administration of glucose and admission on the NICU. The difference in weight between first prenatal visit and the time of the OGTT was calculated as early weight gain. Total gestational weight gain was calculated as the difference in weight between first prenatal visit and delivery. Excessive total gestational weight gain (EGWG) was defined according to the 2009 National Academy of Medicine [NAM, former Institute of Medicine (IOM)] guidelines (39).

### 2.4 Statistical analysis

Descriptive statistics were presented as frequencies and percentages for categorical variables and means with standard deviations or medians with interquartile range for continuous variables. Categorical variables were analyzed using the Chi-square test or the Fisher exact test in case of low (<5) cell frequencies, whereas continuous variables were analyzed using the Kruskal-Wallis test for data with a non-normal distribution or One-way ANOVA test for data with a normal distribution.

Women were divided into groups according to the lowest glucose value measured during the 75g OGTT. To estimate crude and adjusted odds ratios (aORs) of the effects of lowest group of glycemia (<3.9mmol/L) versus the highest group of glycemia (>4.4mmol/L) during the 75g OGTT on delivery outcomes, a conditional logistic regression was used for binary outcomes. Excessive weight gain, inadequate weight gain (less than recommended weight gain according to NAM guidelines), induction of labor, caesarean sections (CS) and LGA were corrected for the following confounding factors: maternal age, ethnic background, smoking during pregnancy, history of macrosomia, multiparity, total gestational weight gain and for early pregnancy BMI, fasting glycemia, fasting insulin, fasting HDL-cholesterol and fasting LDL-cholesterol. Macrosomia and emergency CS were corrected for age, ethnic background, total gestational weight gain, and for early pregnancy BMI, fasting glycemia, and fasting LDL-cholesterol. Gestational hypertension, preterm delivery and low weight infants <2.5kg were corrected for BMI in early pregnancy and total gestational weight gain, while birth weight baby ≥4.5Kg was corrected for BMI in early pregnancy. A Spearman’s correlation test was used to determine the relationship between birth weight and the lowest glycemia at fasting, 1-hour or 2-hour during the 75g OGTT. Logistic regression analysis was performed for the binary outcomes (birth weight <2.5kg and preterm delivery) and fasting glucose or 2-hour post load glucose as continuous predictors. Results are presented as odds ratios with 95% confidence intervals. A p-value <0.05 was considered significant. In addition, a receiver operating characteristic (ROC)-analysis was performed with an estimated area under the curve (AUC) with 95% CI for the binary outcome as response and the continuous predictor as explanatory variable. This analysis results in a sensitivity and specificity level associated with each outcome. The AUC ranges between 0.5 (discrimination no better than chance) and 1 (perfect discrimination). The optimal cut-off value can be selected as the best combination of sensitivity and specificity. If equal importance is given to sensitivity and specificity, the maximum Youden index indicates the best cut-off value. The Youden index was calculated as the sum of sensitivity and specificity minus 1 and ranges from -1 through 1. Analyzes were performed by statistician A. Laenen using SAS software.

## 3 Results

**Figure 1:**
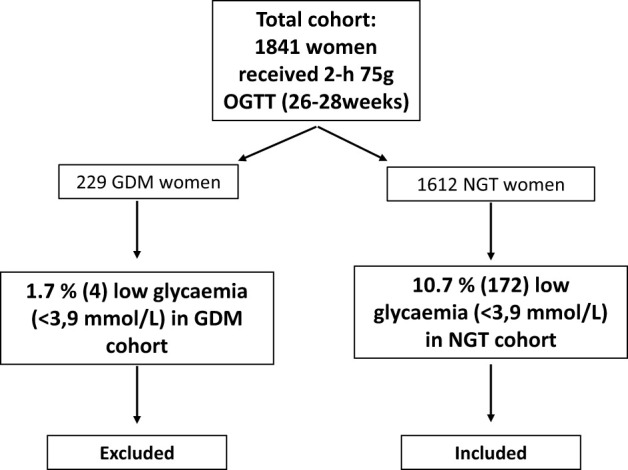
Flowchart in- and exclusion criteria. OGTT, oral glucose tolerance test; GDM, gestational diabetes mellitus; NGT, normal glucose tolerant.

Of the total cohort, 1841 women received a 2-hour 75g OGTT at 26-28 weeks of pregnancy. In the total cohort (NGT and GDM women combined), 9.6% (176) women had a low glycemic value (<3.9 mmol/L) at fasting, 1-hour or 2-hour measurement during the OGTT. Because only four women with GDM had low glycemia during the OGTT, women with GDM were excluded for further analysis (**Figure 1**). Within the NGT-cohort, 10.7% (172) had low glycemia (<3.9 mmol/L), 2.3% (35) had glycemia <3.5 mmol/L and 0.7% (11) had glycemia <3.0 mmol/L at fasting, 1-hour or 2-hour OGTT. Most women (71.5%, n=123) had a low glycemia (<3.9 mmol/L) fasting, 9.3% (16) had a low glycemia at the 1 hour and 27.3% (47) at the 2-hour measurement. Of all NGT women, only 0.6% (10) had a low glycemia at several time points during the OGTT.

### 3.1 Characteristics and pregnancy outcomes of women with low glycemia (<3.9 mmol/L) during the OGTT

Compared to women with glycemia ≥3.9 mmol/L (n=1440, 89.3%), women with low glycemia [<3.9 mmol/L, n=172 (10.7%)] during the OGTT were younger, had a better metabolic profile with a lower BMI, a lower HOMA-IR in early pregnancy and at the time of the OGTT, and less impaired beta-cell function [ISSI-2: 3.3 (2.5-4.0) vs. 2.3 (1.9-2.8); p<0.0001] at the time of the OGTT (**Table 1**). Women with low glycemia had more often inadequate gestational weight gain (less than recommended by NAM) and less labor inductions compared to women with higher glucose values during the OGTT (**Table 1**). There were no differences in diet score or physical activity between both groups (**Table 1**). Women with a low glycemia during the OGTT had also more often low glycemia (<3.9mmol/L) and hypoglycemia (<3.5 mmol/L) at the non-fasting glycemia measurement before the GCT at 24-26 weeks of pregnancy [respectively 19.6% (33) vs. 6.6% (92); p<0.001 and 7.7% (13) vs. 1.7% (24); p<0.001] (**Table 1**).

**Table 1:** Comparison of characteristics and pregnancy outcomes between women with glycemia <3.9 mmol/L and women with glycemia ≥3.9 mmol/L at fasting, 1h or 2h 75g OGTT in the normal glucose tolerance group.

NGT-group			
	Glycemia <3.9mmol/L N=172 (10.7%)	Glycemia ≥3.9 mmol/L N=1440 (89.3%)	P-value
General			
Age (years)	29.9 ± 3.9	30.7 ± 3.9	**0.007**
% Ethnic minorities	6.4 (11)	8.5 (121)	0.363
% Multiparity	45.3 (78)	46.5 (670)	0.769
6-14 weeks visit			
Week first visit with FPG	12.2 ± 1.5	11.9 ± 1.8	**0.024**
BMI (Kg/m²) % BMI <18.5 % BMI 18.5-24.9	22.7 ± 3.9 4.1 (7) 76.6 (131)	24.6 ± 4.5 2.6 (37) 26.2 (375)	**<0.001**
% Overweight % Obesity	19.3 (33) 5.8 (10)	37.9 (542) 11.7 (167)	**<0.001** **0.022**
% Waist ≥80cm	62.1 (100)	75.5 (1044)	**<0.001**
Weight gain (first visit till OGTT) (Kg)	6.8 ± 2.7	7.2 ± 3.4	0.071
Systolic blood pressure (mmHg)	113.6 ± 9.8	115.0 ± 10.5	0.098
Diastolic blood pressure (mmHg)	68.2 ± 7.1	70.6 ± 8.2	**<0.001**
Fasting glycemia (mmol/L)	4.3 (4.1-4.4)	4.6 (4.3-4.7)	**<0.001**
HOMA-IR	1.0 (0.7-1.4)	1.3 (1.0-1.9	**<0.001**
HOMA-B	133.7 (92.6-204.3)	131.8 (96.3-183.3)	0.482
HbA1c (mmol/mol and %)	30 (28-32) 4.9 (4.7-5.1)	31 (29-32) 5.0 (4.8-5.1)	**<0.001**
Fasting Total cholesterol (mmol/L)	4.6 (4.1-5.2)	4.7 (4.2-5.2)	0.171
Fasting HDL (mmol/L)	1.8 (1.6-2.1)	1.7 (1.5-2.0)	**0.011**
Fasting LDL (mmol/L)	2.3 (1.9-2.7)	2.4 (2.0-2.9)	**0.006**
Fasting TG (mmol/L)	1.0 (0.8-1.1)	1.0 (0.8-1.3)	0.518
Total Score lifestyle Physical activity Diet	1.0 (0.0-2.0) 2.0 (0.0-5.0)	1.0 (0.0-2.0) 2.0 (0.0-4.0)	0.446 0.215
24-28 weeks visit			
BMI (Kg/m²) % BMI <18.5 % BMI 18.5-25	25.2 ± 3.8 1.2 (2) 60.6 (100)	27.2 ± 4.4 0.1 (1) 36.0 (506)	**<0.001**
Systolic blood pressure (mmHg)	111.9 ± 10.5	113.3 ± 10.0	0.092
Diastolic blood pressure (mmHg)	65.9 ± 7.9	67.1 ± 7.9	**0.026**
Glucose non-fasting 0 min on GCT (mmol/L)	4.5 ± 0.7	4.9 ± 0.9	**<0.001**
% Glucose <3.9 mmol/L non-fasting 0min on GCT	19.6 (33)	6.6 (92)	**<0.001**
% Glucose <3.5 mmol/L non-fasting 0min on GCT	7.7 (13)	1.7 (24)	**<0.001**
Glucose 60 min on GCT (mmol/L)	6.2 ± 1.4	6.5 ± 1.4	**0.002**
% Glucose <3.9 mmol/L 60min on GCT	2.3 (4)	1.7 (24)	0.533
% Glucose <3.5 mmol/L 60min on GCT	0.6 (1)	0.7 (10)	1.000
Fasting glycemia (mmol/L)	3.8 (3.7-3.9)	4.3 (4.2-4.6)	**<0.001**
30 min glucose OGTT (mmol/L)	6.3 (5.6-7.1)	6.9 (6.3-7.7)	**<0.001**
1-hour glucose OGTT (mmol/L)	6.0 (4.8-7.0)	6.9 (6.0-7.9)	**<0.001**
2-hour glucose OGTT (mmol/L)	4.9 (3.8-6.0)	6.0 (5.3-6.9)	**<0.001**
HbA1c (mmol/mol and %)	29 (27-30) 4.8 (4.6-4.9)	30 (29-32) 4.9 (4.8-5.1)	**<0.001**
Matsuda insulin sensitivity	0.8 (0.6-1.1)	0.6 (0.4-0.8)	**<0.001**
HOMA-IR	1.2 (0.8-1.7)	1.8 (1.3-2.5)	**<0.001**
HOMA-B	409.5 (237.2-619.5)	220.8 (160.7-309.9)	**<0.001**
ISSI-2	3.3 (2.5-4.0)	2.3 (1.9-2.8)	**<0.001**
Insulinogenic index/HOMA-IR	0.5 (0.3-0.7)	0.3 (0.2-0.4)	**<0.001**
Fasting Total cholesterol (mmol/L)	6.2 (5.5-7.2)	6.3 (5.7-7.0)	0.795
Fasting HDL (mmol/L)	2.0 (1.6-2.4)	1.9 (1.6-2.2)	0.057
Fasting LDL (mmol/L)	3.4 (2.8-4.2)	3.4 (2.9-4.2)	0.853
Fasting TG (mmol/L)	1.7 (1.4-2.2)	1.8 (1.4-2.3)	0.147
Total score lifestyle Physical activity Diet	1.5 (0.0-2.0) 2.0 (0.0-5.0)	1.0 (0.0-2.0) 2.0 (0.0-4.0)	0.506 0.172
IPAQ low	13.9 (23)	16.6 (229)	0.373
Delivery			
Total Weight gain (first visit till delivery) (Kg)	11.3 ± 4.1	12.3 ± 5.1	**0.005**
% Excessive weight gain	15.3 (20)	29.7 (363)	**<0.001**
% Inadequate weight gain	51.1 (67)	29.7 (363)	**<0.001**
Gestational age (weeks)	39.0 ± 1.7	39.3 ± 1.6	**0.011**
% Preeclampsia	1.7 (3)	1.8 (26)	1.000
% Gestational hypertension	2.3 (4)	4.4 (64)	0.232
% Preterm delivery	7.0 (12)	5.2 (74)	0.323
% Induction labor	16.3 (28)	27.1 (388)	**0.002**
% Caesarean sections (total)	19.2 (33)	20.3 (291)	0.723
Weight baby (g)	3286.3 ± 534.4	3411.3 ± 505.4	**0.004**
% Weight baby <2.5 kg	5.8 (10)	3.9 (56)	0.225
% Macrosomia (>4Kg)	6.4 (11)	9.8 (140)	0.149
% LGA	9.9 (17)	13.2 (189)	0.227
% SGA	7.6 (13)	4.8 (68)	0.110
% Shoulder dystocia	0.6 (1)	1.2 (17)	0.712
% Neonatal hypoglycemia <2.2 mmol/L	2.8 (3)	4.1 (38)	0.514
% NICU admission	8.2 (14)	9.7 (139)	0.528

OGTT, oral glucose tolerance test; GCT, glucose challenge test; GDM, gestational diabetes mellitus; BMI, Body Mass Index; HOMA-IR, Homeostatic Model Assessment for Insulin Resistance; HOMA-B,, Homeostatic Model Assessment for B-cell secretion; ISSI-2, Insulin Secretion-Sensitivity Index-2 HDL, high-density lipoprotein; LDL, low-density-lipoprotein; TG, triglycerides; IPAQ, International Physical Activity Questionnaire; LGA, large-for-gestational age infant; SGA, small-for-gestational age infant; NICU, neonatal intensive care unit; IFG, impaired fasting glycemia; IGT, impaired glucose tolerance; Overweight, BMI ≥25-29.9 Kg/m²; Obesity, BMI ≥30 Kg/m. Categorical variables are presented as frequencies %(n); continuous variables are presented as mean ± SD if normally distributed and as median ± IQR if not normally distributed; Differences are considered significant at p-value<0.05. Bold means a statistical significant value of p<0.05.

### 3.2 Characteristics of women with hypoglycemia (<3.5 mmol/L) during the OGTT

Women with a value <3.5 mmol/L (2.3%, n=35) during the OGTT were younger, were more often single and smoked less often before pregnancy compared to women with a glycemia ≥3.5 mmol/L (**Appendix I**). Women with value <3.5 mmol/L during the OGTT had also more often low glycemia (<3.9mmol/L) and glycemia <3.5mmol/L at the non-fasting glycemia measurement before the GCT [respectively 22.9% (8) vs. 7.6% (117); p=0.005 and 11.4% (4) vs. 2.1% (33); p=0.008]. At the time of the OGTT, these women were less insulin resistant (lower HOMA-IR), had a less impaired beta-cell function (higher ISSI-2 index), and had also more often inadequate gestational weight gain compared to women with glycemia ≥3.5 mmol/L. There were no differences in diet score or physical activity, nor differences in pregnancy outcomes between both groups (**Appendix I**).

### 3.3 Characteristics and pregnancy outcomes of women in the lowest quartile group (<3.9 mmol/L) compared to the highest quartile group (>4.4 mmol/L) of low glucose levels during the OGTT

Women in the lowest quartile group (10.7%, n=172) of glycemia (<3.9 mmol/L) measured during the OGTT were younger and had more often a paid job compared to women (29.9%, n=482) in the highest quartile group (glycemia >4.4 mmol/L) (**Table 2**). Overview of the four quartile groups is available in **Appendix II**. There were no differences in alcohol consumption or smoking before and during pregnancy between the lowest and highest quartile groups. In early pregnancy and at the time of the OGTT, women in the lowest quartile group had a lower BMI, were less insulin resistant (lower HOMA-IR) and had a less impaired beta-cell function (higher ISSI-2 index) compared to the highest quartile group (**Table 2**). Of all women in the lowest quartile group, 10.5% (18) had also a glycemia <3.9 mmol/L in early pregnancy compared to none of the women in the highest quartile group (p<0.001). Women in the lowest quartile group had also more often low glycemia (<3.9 mmol/L) and glycemia <3.5 mmol/L at the non-fasting glycemia measurement before the GCT [respectively 19.6% (33) vs. 2.7% (13); p<0.001 and 7.7% (13) vs. 0.6% (3); p<0.001] compared to women in the highest quartile group of glycemia during the OGTT. There was no difference in gestational weight gain between the first perinatal visit and the time of the OGTT. However, at the time of delivery, women in the lowest quartile group had more often less gestational weight gain than recommended [51.1% (67) vs. 29.5% (123); p<0.001] compared to women in the highest quartile group. There were no differences in diet score or physical activity between both groups (**Table 2**). Women in the lowest quartile group, had less often gestational hypertension and less need for labor inductions or emergency CS, but had more often infants with a birth weight <2.5Kg [5.8% (10) vs. 1.9% (9); p=0.009] compared to women with glycaemia >4.4 mmol/L (**Table 2**). However, within the group with neonates with low birth weight, there was no difference in rates of SGA, preterm delivery or intra-uterine growth restriction. The lower rate of labor inductions [aOR 0.54, 95% CI (0.30-0.96); p=0.036] and the increased rate of infants with low birth weight [aOR 3.41, 95% CI (1.17-9.92); p=0.025] remained significant after adjustments for confounders (**Table 3**). A birth weight <2.5Kg occurred also twice as frequently in the 2^nd^ group (27.4%, n=441, 3.9-4.2 mmol/L) and 3^rd^ group (32.1%, n=517, 4.25-4.4 mmol/L) compared to the highest quartile glycemia group [respectively 5.0% (22) vs. 1.9% (9); p=0.009 and 4.9% (25) vs. 1.9% (9); p=0.010] (**Appendix II**). This remained significant after adjustment for confounders [respectively, aOR 2.69, 95% CI (1.06-6.80); p=0.037 and aOR 3.25, 95% CI (1.33-7.97); p=0.010] compared to the highest quartile glycemia group.

**Table 2:** Comparison of characteristics and pregnancy outcomes between women with glycemia <3.9 mmol/L (lowest quartile) and women with glycemia >4.4 mmol/L (highest quartile) at fasting, 1h or 2h 75g OGTT in the normal glucose tolerance group.

NGT-group			
	Glucose <3.9 mmol/L N= 172 (10.7%)	Glucose >4.4 mmol/L N=482 (29.9%)	P-value
General			
Age (years)	29.9 ± 3.9	31.1 ± 4.1	**<0.001**
% Ethnic minorities	6.4 (11)	10.9 (52)	0.091
% multiparity	45.3 (78)	50.8 (245)	0.217
% paid job	94.1 (161)	89.0 (427)	**0.049**
% living without partner	17.1 (29)	20.2 (97)	0.378
% smoking before pregnancy	25.3 (43)	29.2 (140)	0.335
% smoking during pregnancy	2.9 (5)	4.6 (22)	0.350
% Alcohol use before pregnancy	69.5 (119)	66.3 (317)	0.241
% Alcohol use during pregnancy	8.2 (14)	6.3 (30)	0.596
% First degree family history of diabetes	8.8 (14)	10.4 (48)	0.571
% History of GDM*	5.1 (4)	6.6 (16)	0.649
% History of macrosomia >4Kg*	4.1 (7)	7.9 (38)	0.172
6-14 weeks visit			
BMI (Kg/m²)	22.7 ± 3.9	25.9 ± 5.0	**<0.001**
% Underweight % Overweight % Obesity	4.1 (7) 19.3 (33) 5.8 (10)	2.1 (10) 51.7 (246) 17.6 (84)	0.162 **<0.001** **<0.001**
% Waist ≥80cm	62.1 (100)	81.1 (374)	**<0.001**
Systolic blood pressure (mmHg)	113.6 ± 9.9	116.0 ± 10.8	**0.008**
Diastolic blood pressure (mmHg)	68.2 ± 7.1	71.3 ± 8.6	**<0.001**
Fasting glycemia (mmol/L)	4.3 (4.1-4.4)	4.7 (4.6-4.8)	**<0.001**
Fasting glycemia <3.9 mmol/L	10.5 (18)	0 (0)	**<0.001**
HOMA-IR	1.0 (0.7-1.4)	1.6 (1.2-2.2)	**<0.001**
HOMA-B	133.7 (92.6-204.3)	124.8 (93.6-178.4)	0.243
HbA1c (mmol/mol and %)	30 (29-32) 4.9 (4.7-5.1)	31 (30-33) 5.0 (4.9-5.2)	**<0.001**
Fasting Total cholesterol (mmol/L)	4.6 (4.1-5.2)	4.7 (4.2-5.3)	0.042
Fasting HDL (mmol/L)	1.8 (1.5-2.1)	1.7 (1.5-1.9)	**<0.001**
Fasting LDL (mmol/L)	2.3 (1.9-2.7)	2.5 (2.0-2.9)	**<0.001**
Fasting TG (mmol/L)	1.0 (0.8-1.1)	1.0 (0.8-1.3)	**0.040**
Total Score lifestyle Physical activity Diet	1.0 (0.0-2.0) 2.0 (0.0-5.0)	1.0 (0.0-2.0) 2.0 (-1.0-4.0)	0.062 0.176
Weight gain (first visit till OGTT) (Kg)	6.8 ± 2.7	7.1 ± 3.4	0.315
24-28 weeks visit			
BMI (Kg/m²)	25.2 ± 3.8	28.5 ± 4.8	**<0.001**
Systolic blood pressure (mmHg)	111.9 ± 10.5	114.4 ± 10.4	**0.006**
Diastolic blood pressure (mmHg)	65.9 ± 7.9	68.7 ± 7.8	**<0.001**
Glucose non-fasting 0 min on GCT (mmol/L)	4.5 ± 0.8	5.1 ± 0.9	**<0.001**
% Glucose <3.9 mmol/L non-fasting 0min on GCT	19.6 (33)	2.7 (13)	**<0.001**
% Glucose <3.5 mmol/L non-fasting 0min on GCT	7.7 (13)	0.6 (3)	**<0.001**
Glucose 60 min on GCT (mmol/L)	6.2 ± 1.4	6.6 ± 1.4	**<0.001**
% Glucose <3.9 mmol/L 60min on GCT	2.3 (4)	0.8 (4)	0.128
% Glucose <3.5 mmol/L 60min on GCT	0.6 (1)	0.4 (2)	0.785
Fasting glycemia (mmol/L)	3.8 (3.7-3.9)	4.7 (4.6-4.8)	**<0.001**
30 min glucose OGTT (mmol/L)	6.3 (5.6-7.1)	7.2 (6.7-7.9)	**<0.001**
1-hour glucose OGTT (mmol/L)	6.0 (3.8-6.0)	7.3 (6.4-8.3)	**<0.001**
2-hour glucose OGTT (mmol/L)	4.9 (3.8-6.0)	6.3 (5.5-7.1)	**<0.001**
HbA1c (mmol/mol and %)	29 (28-30) 4.8 (4.6-4.9)	31 (29-32) 5.0 (4.8-5.1)	**<0.001**
Matsuda insulin sensitivity	5.9 (4.1-7.6)	3.1 (2.4-4.2)	**<0.001**
HOMA-IR	1.2 (0.8-1.7)	2.4 (1.7-3.1)	**<0.001**
HOMA-B	409.5 (237.2-619.5)	189.9 (141.1-260.4)	**<0.001**
ISSI-2	3.3 (2.5-4.0)	1.9 (1.7-2.3)	**<0.001**
Insulinogenic index/HOMA-IR	0.5 (0.3-0.8)	0.2 (0.2-0.3)	**<0.001**
Fasting Total cholesterol (mmol/L)	6.2 (5.5-7.2)	6.3 (5.7-7.1)	0.852
Fasting HDL (mmol/L)	2.0 (1.6-2.4)	1.9 (1.6-2.1)	**<0.001**
Fasting LDL (mmol/L)	3.4 (2.8-4.2)	3.5 (2.9-4.2)	0.689
Fasting TG (mmol/L)	1.7 (1.4-2.2)	1.9 (1.5-2.4)	**0.002**
Total score lifestyle Physical activity Diet	1.5 (0.0-2.0) 2.0 (0.0-5.0)	1.0 (0.0-2.0) 2.0 (-1.0-4.0)	0.226 0.052
% IPAQ low	13.9 (23)	17.1 (79)	0.336
Delivery			
Total Weight gain (first visit till delivery) (Kg)	11.3 ± 4.1	12.0 ± 5.6	**0.062**
% excessive weight gain	15.3 (20)	33.8 (141)	**<0.001**
% inadequate weight gain	51.1 (67)	29.5 (123)	**<0.001**
Gestational age (weeks)	39.0 ± 1.7	39.4 ± 1.5	**0.026**
% Preeclampsia	1.7 (3)	2.3 (11)	0.682
% Gestational hypertension	2.3 (4)	6.8 (33)	**0.029**
% Preterm delivery	7.0 (12)	4.2 (20)	0.143
% Induction labor	16.3 (28)	32.4 (156)	**<0.001**
% Caesarean sections (total)	19.2 (33)	24.3 (117)	0.127
% Emergency CS (during labor)	7.6 (13)	13.3 (64)	**0.045**
Weight baby (g)	3286.3 ± 534.4	3468.6 ± 515.6	**<0.001**
% Weight baby <2.5 kg Of which: % SGA % preterm delivery % Intrauterine growth restriction	5.8 (10) 60.0 (6) 40.0 (4) 0 (0)	1.9 (9) 33.3 (3) 55.6 (5) 0 (0)	**0.009** 0.484 0.632 -
% Macrosomia (>4Kg)	6.4 (11)	12.7 (61)	**0.023**
% Weight baby ≥4.5Kg	0.6 (1)	2.3 (11)	0.151
% LGA	9.9 (17)	17.1 (82)	**0.026**
% SGA	7.6 (13)	4.2 (20)	0.079
%Shoulder dystocia	0.6 (1)	0.8 (4)	0.744
% Neonatal hypoglycemia <2.2 mmol/L	2.8 (3)	4.7 (15)	0.404
% NICU admission	8.2 (14)	9.8 (47)	0.535

OGTT, oral glucose tolerance test; GCT, glucose challenge test; GDM, gestational diabetes mellitus; BMI, Body Mass Index; HOMA-IR, Homeostatic Model Assessment for Insulin Resistance; HOMA-B,, Homeostatic Model Assessment for B-cell secretion; ISSI-2, Insulin Secretion-Sensitivity Index-2 HDL, high-density lipoprotein; LDL, low-density-lipoprotein; TG, triglycerides; IPAQ, International Physical Activity Questionnaire; LGA, large-for-gestational age infant; SGA, small-for-gestational age infant; NICU, neonatal intensive care unit; IFG, impaired fasting glycemia; IGT, impaired glucose tolerance; Overweight, BMI ≥25-29.9 Kg/m²; Obesity, BMI ≥30 Kg/m. Categorical variables are presented as frequencies %(n); continuous variables are presented as mean ± SD if normally distributed and as median ± IQR if not normally distributed; Differences are considered significant at p-value<0.05. *A history of GDM and a history of a macrosomic baby were calculated on the number of women with a previous pregnancy. Bold means a statistical significant value of p<0.05.

**Table 3:** Adjusted odds ratios for pregnancy outcomes comparing the lowest quartile of low glycemia (<3.9mmol/L) with the highest quartile of low glycemia (>4.4mmol/L) during the 75g OGTT.

Outcome	Crude OR	95%CI	P-value	Adjusted OR	95%CI	P-value
% Excessive weight gain*	0.35	0.21-0.59	<0.001	0.65	0.21-1.99	0.451
% Inadequate weight gain*	2.50	1.67-3.74	<0.001	1.00	0.37-2.71	0.995
% Gestational hypertension***	0.33	0.11-0.9.	0.037	0.41	0.12-1.40	0.155
% Preterm delivery***	1.72	0.82-3.61	0.148	1.734	0.73-4.09	0.208
% Labor induction *	**0.40**	**0.26-0.63**	**<0.001**	**0.64**	**0.30-0.96**	**0.036**
% Emergency CS **	0.53	0.29-0.99	0.048	0.45	0.18-1.16	0.099
% Weight baby <2.5 kg***	**3.22**	**1.29-8.07**	**0.012**	**3.41**	**1.17-9.92**	**0.025**
% Macrosomia (>4Kg)**	0.47	0.24-0.91	0.026	0.68	0.29-1.63	0.392
% LGA*	0.54	0.31-0.93	0.027	0.81	0.38-1.74	0.589

OR: odds ratio; CI: confidence interval; LGA: large-for-gestational age infant. Differences are considered significant at p-value<0.05.

* Adjusted for age, ethnic minority background, smoking during pregnancy, history of macrosomia, multiparity, BMI in early pregnancy, fasting glycemia in early pregnancy, fasting insulin in early pregnancy, fasting HDL)cholesterol in early pregnancy, fasting LDL-cholesterol in early pregnancy and total gestational weight gain.

** Adjusted for age, ethnic minority, BMI in early pregnancy, fasting glycemia in early pregnancy, fasting LDL-cholesterol in early pregnancy and total gestational weight gain.

*** Adjusted for BMI in early pregnancy and total gestational weight gain. Bold means a statistical significant value of p<0.05.

**Figure 2:**
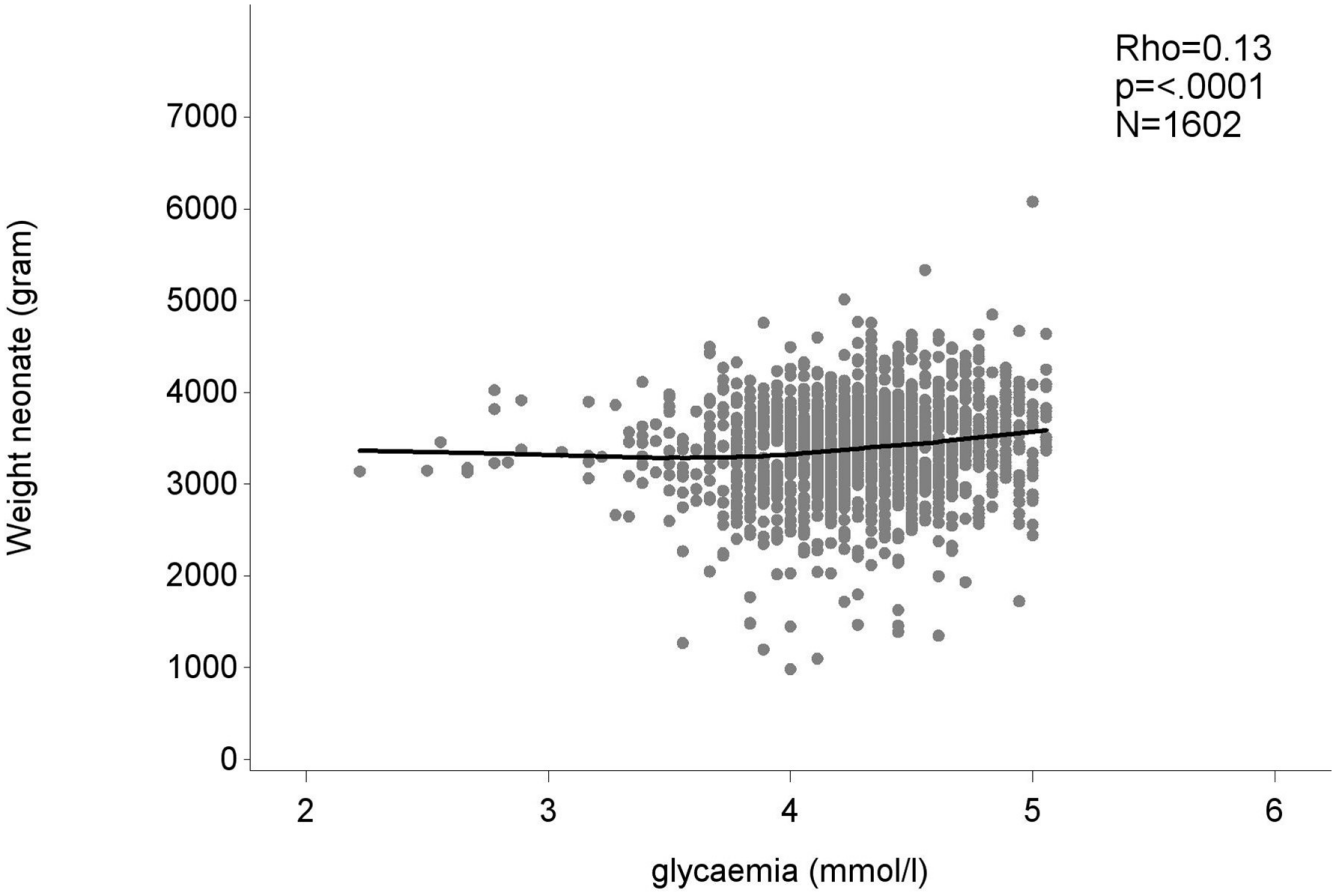
The association between the weight of the neonate and the lowest glycemia value measured at fasting, 1- or 2-hour measurement of the 75g OGTT. Weight neonate in gram, glycemia in mmol/l.

**Figure 3:**
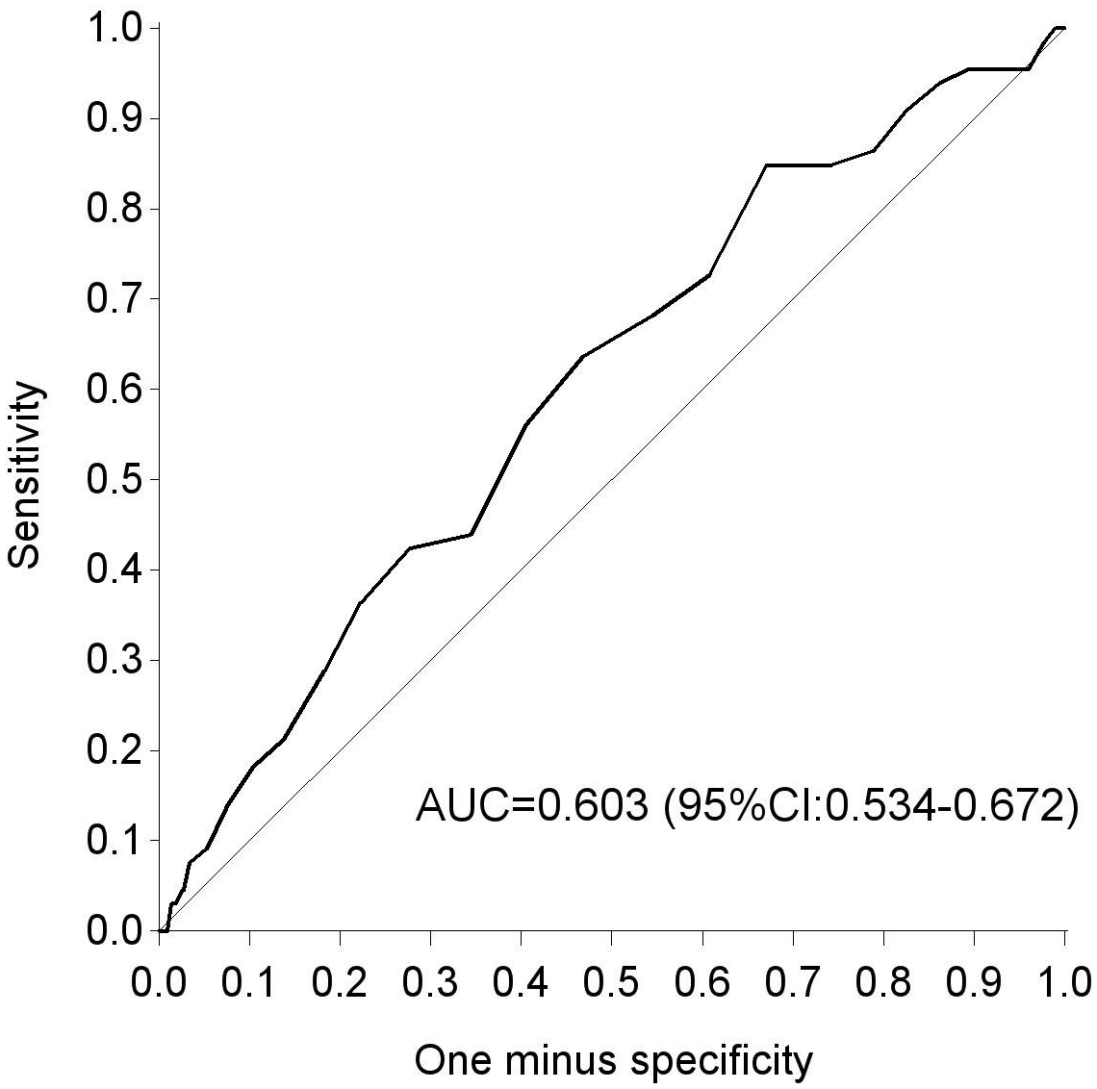
ROC curve for fasting glycemia during 75g OGTT as a predictor for a birth weight <2.5 kg. AUC, area under the curve; ROC, receiver-operating curve.

There was a weak positive correlation between birth weight and glycemia during the OGTT [r(1600) = 0.13; p<0.001] (**Figure 2**). As fasting glycemia decreased, the risk for a low birth weight increased. An estimation of the cut-off for fasting glycemia during the OGTT with best trade-off between sensitivity and specificity (with maximum Youden index) to predict a low birth weight <2.5Kg, was seen at a FPG of 4.4mmol/L, with a sensitivity of 84.8% and specificity of 32.9% (**Appendix III**). The AUC on the ROC curve for fasting glycemia as a predictor for low birth weight (<2.5kg) was 0.603 (95% CI 0.534-0.672) (**Figure 3**).

## 4 Discussion

We found that 10.7% of NGT women had a low glycemic value (<3.9 mmol/L) during the 75g OGTT, most often at the fasting measurement. This is in line with a Turkish study reporting a prevalence of 11.4%, using the same cut-off of 3.9 mmol/L (ADA level for hypoglycemia outside pregnancy) measured during a 75g OGTT (7). In our study, only 2.3% of NGT women had a glycemic value <3.5 mmol/L during the 75g OGTT. This is less than reported by other studies (12–14), however cut-offs for hypoglycemia differed among the different studies.

To the best of our knowledge, we are the first to report that compared to women with glycemia values >4.4 mmol/L during the OGTT, women with glycemia <3.9 mmol/L, had a better metabolic profile with a lower BMI, less insulin resistance, and less impaired beta-cell function, but higher rates of a birth weight <2.5 Kg with an aOR of 3.41. Importantly, this increased risk remained significant after adjustment for confounders such as BMI and total gestational weight gain since women with low glycemia gained more often less weight than recommended. In addition, we excluded women with GDM, which has the advantage that in our study women did not receive any treatment influencing glycemia. Our results are in line with the Turkish study, in which they also found a higher rate of low-birth-weight neonates in women with low glycemia defined as <3.9 mmol/L (or 70 mg/dL) (7).

An association between a low birth weight and low glycemia or reactive hypoglycemia has been reported by other studies focusing on GDM-women, often using different cut-offs for glycemia (for example <2.8 mmol/L) or a different glucose load for the OGTT (for example 100g OGTT) (10, 13, 14, 17).

In pregnant women with pregestational diabetes and in women with GDM, it has been clearly demonstrated that hyperglycemia increases the risk for macrosomia and LGA infants, since the fetus is dependent on nutrients of the mother and higher glucose levels in the fetus lead to fetal hyperinsulinism (40). However, less data are available on the potential effects of low glycemia on pregnancy outcomes (40). A study with 334 women with GDM, who were matched for obesity, race and parity, showed that the rate of SGA was significantly higher in the low glycemia (<4.8 mmol/L) group compared to the non-diabetic control group (41). Our results also showed that fasting glycemia during the 75g OGTT can be a predictor for a low birth weight, as the risk for this outcome increases when fasting glycemia decreases. Exploratory analysis on our data showed an AUC of 0.603 (95% CI 0.534-0.672) for a fasting cut-off of 4.4 mmol/L, indicating that this has only a poor predictive value for a low birth weight. In addition, our logistic regression result suggested a correlation between a glycaemic value of 3.9 mmol/L and low birth weight, which is stronger related with the point of 3.5 mmol/L. This is in line with a recent study in the UK, which showed that fasting glycemia or a 2-hour postload glycemia <3.5mmol/L during the 75g OGTT can be a predictor for low birth weight (17). However, this study focused on a high risk population with GDM, whereas our study only included NGT women.

Our results also indicate that women with low glycemia during the OGTT, had significantly more often already low glycemia in early pregnancy and a low non-fasting glycemia in the weeks before the OGTT, suggesting that these women have more often a lower glycemia throughout pregnancy. Previous studies have reported associations between hypoglycemia on the 50g GCT and SGA infants (42, 43). This association was mainly seen on the 1-hour GCT value, which is in contrast to our results since we only found an association with the non-fasting random glucose measured before the GCT. Our results also indicate that the increased risk for a low birth weight is independent of confounders such as BMI and inadequate gestational weight gain. In addition, there were also no differences in diet nor in physical activity between the different groups in our study. This suggests that a low glycemia during pregnancy might be a marker of placental insufficiency (7, 8). It is known that less severe deficiencies in arterial remodeling of the placenta result in SGA infants (44, 45). In addition, if maternal blood glucose decreases, less glucose is transferred to the fetus, leading to lower insulin production by the fetus, which might lead to growth restriction (4).However, there is currently no evidence from intervention studies that a more strict follow-up or different management strategy for women with low glycemia during the OGTT, might reduce the risk to deliver an infant with low birth weight. In addition, not only prevention of SGA infants is important, as infants with a low birth weight (<2.5 Kg) as such are also at increased risk to develop T2DM and cardiovascular disease later in life. This increased risk for an adverse metabolic profile later in life, might be related to adaptations by the fetus induced by the lower glucose levels, leading to abnormal pancreatic beta-cell function and reduced capacity to secrete insulin extending into adult life (4). Additionally, insulin secretion and insulin resistance might also be genetically determined and as such affect intrauterine growth (3, 4).

A major strength of our study is the large multicentric prospective cohort with a large, detailed dataset containing broad demographic, clinical and obstetrical outcomes. We provide the first data on the association between both maternal and neonatal outcomes in NGT women with low glycemia measured fasting or at the 1-hour or 2-hour time point during a 75g OGTT. Data on the risk for adverse pregnancy outcomes were adjusted for important confounders. In addition, women with GDM were excluded, so that we could evaluate pregnancy outcomes in a non-treated population. We used fluoride-oxalate tubes to collect blood samples for the analyses of glucose, limiting the risk for false low glucose values as fluoride inhibits glycolysis. The blood samples were also sent immediately to the laboratory for analyzes. Furthermore, glycemia was analyzed at different time points during pregnancy (at 11 weeks, 24-26 weeks and 26-28 weeks). A limitation of the study is the mainly Caucasian population in our cohort. In addition, we had no detailed data on nutrition from food diaries and we had no follow-up data on the evolution of glycemia after the OGTT in pregnancy. As the group with low postload glycemia was small, differences in pregnancy outcomes between women with low fasting glycemia and women with low postload glycemia could not be adequately evaluated. We had also no data on placental blood flow to evaluate placental insufficiency.

## 5 Conclusion

In conclusion, our results suggest that women with a glycemic value (<3.9 mmol/L) during the 75g OGTT are at increased risk to deliver an infant with a low birth weight (<2.5Kg). Importantly, this increased risk remained significant after adjustment for confounders such as BMI and low gestational weight gain.

## Data availability statement

The raw data supporting the conclusions of this article will be made available by the authors, without undue reservation.

## Ethics statement

The studies involving human participants were reviewed and approved by University Hospitals Leuven, Leuven, Belgium. The patients/participants provided their written informed consent to participate in this study.

## Author contributions

KB conceived the sub-analysis. LR and LD prepared the data and ALa did the statistical analysis. LR did the literature review. LR and KB wrote the first draft of the manuscript. All authors contributed to the article and approved the submitted version. The corresponding author LR had full access to all the data in the study and had final responsibility for the contents of the article and the decision to submit for publication.
