# Supplementary material for: Normal glucose tolerant women with low glycemia during the oral glucose tolerance test have a higher risk to deliver a low birth weight infant
Source: Front Endocrinol (Lausanne). 2023 Jun 2;14:1186339. doi: 10.3389/fendo.2023.1186339 (PMC10272607; doi:10.3389/fendo.2023.1186339)
Supplement: Supplementary file 1 [file Table_1.docx]

**Appendix I: Comparison of characteristics and pregnancy outcomes between women with glycemia <3.5 mmol/L and women with glycemia ≥3.5 mmol/L at fasting, 1h or 2h 75g OGTT in the normal glucose tolerance group**

| NGT-group | | | |
| --- | --- | --- | --- |
|  | Glycemia <3.5 mmol/L  N=35 (2.2%) | Glycemia ≥3.5 mmol/L  N=1577 (97.8%) | P-value |
| **General** | | | |
| Age (years) | 29.1 ± 4.0 | 30.7 ± 3.9 | **0.019** |
| % Ethnic minorities | 5.7 (2) | 8.3 (130) | 1.000 |
| % multiparity | 28.6 (10) | 46.8 (738) | **0.039** |
| **6-14 weeks visit** | | | |
| BMI (Kg/m²) | 23.4 ± 4.3 | 24.4 ± 4.5 | 0.100 |
| % Overweight  % Obesity | 20.0 (7)  8.6 (3) | 36.2 (568)  11.1 (174) | 0.051  1.000 |
| % Waist ≥80cm | 65.6 (21) | 74.3 (1123) | 0.307 |
| Weight gain (first visit till OGTT) (Kg) | 6.6 ± 2.3 | 7.1 ± 3.4 | 0.158 |
| Systolic blood pressure (mmHg) | 113.8 ± 9.7 | 114.8 ± 10.4 | 0.495 |
| Diastolic blood pressure (mmHg) | 69.2 ± 5.8 | 70.3 ± 8.1 | 0.602 |
| Fasting glycemia (mmol/L) | 4.3 (4.1-4.6) | 4.5 (4.3-4.7) | **0.002** |
| HOMA-IR | 1.1 (0.8-1.4) | 1.3 (0.9-1.9) | **0.018** |
| HOMA-B | 126.8 (103.2-188.2) | 132.2 (96.1-184.9) | 0.997 |
| HbA1c (mmol/mol and %) | 4.9 (4.8-5.1) | 5.0 (4.8-5.1) | 0.246 |
| Fasting Total cholesterol (mmol/L) | 4.6 (4.0-5.2) | 4.7 (4.2-5.2) | 0.647 |
| Fasting HDL (mmol/L) | 1.8 (1.5-2.2) | 1.7 (1.5-2.0) | 0.302 |
| Fasting LDL (mmol/L) | 2.3 (1.9-2.6) | 2.4 (2.0-2.9) | 0.191 |
| Fasting TG (mmol/L) | 1.0 (0.8-1.1) | 1.0 (0.8-1.2) | 0.919 |
| Total Score lifestyle  Physical activity  Diet | 1.0 (0.0-2.0)  3.0 (0.0-6.0) | 1.0 (0.0-2.0)  2.0 (0.0-4.0) | 0.127  0.199 |
| **24-28 weeks visit** | | | |
| BMI (Kg/m²) | 25.8 ± 4.3 | 27.0 ± 4.4 | 0.068 |
| Systolic blood pressure (mmHg) | 112.3 ± 10.2 | 113.1 ± 10.1 | 0.950 |
| Diastolic blood pressure (mmHg) | 66.5 ± 6.5 | 67.0 ± 7.9 | 0.957 |
| Glucose non-fasting 0 min on GCT (mmol/L) | 4.5 ± 0.8 | 4.9 ± 0.9 | **0.012** |
| % Glucose <3.9 mmol/L non-fasting 0 min on GCT | 22.9 (8) | 7.6 (117) | **0.005** |
| % Glucose <3.5 mmol/L non-fasting 0 min on GCT | 11.4 (4) | 2.1 (33) | **0.008** |
| Glucose 60 min on GCT (mmol/L) | 5.9 ± 1.5 | 6.5 ± 1.4 | **0.006** |
| % Glucose <3.9 mmol/L 60 min on GCT | 2.9 (1) | 1.7 (27) | 0.468 |
| % Glucose <3.5 mmol/L 60 min on GCT | 0 (0) | 0.7 (11) | 1.000 |
| Fasting glycemia (mmol/L) | 3.9 (3.4-4.3) | 4.3 (4.1-4.6) | **<0.001** |
| 1-hour glucose OGTT (mmol/L) | 5.0 (3.4-6.8) | 6.8 (5.9-7.8) | **<0.001** |
| 2-hour glucose OGTT (mmol/L) | 3.4 (3.3-5.0) | 6.0 (5.2-6.9) | **<0.001** |
| HbA1c  (mmol/mol and %) | 29 (28-31)  4.8 (4.7-5.0) | 30 (29-32)  4.9 (4.8-5.1) | **0.013** |
| Matsuda insulin sensitivity | 6.2 (4.3-9.8) | 4.0 (2.9-5.5) | **<0.001** |
| HOMA-IR | 1.3 (0.9-1.9) | 1.7 (1.2-2.4) | **0.004** |
| HOMA-B | 228.7 (-212.9-339.6) | 229.3 (164.1-329.2) | 0.282 |
| ISSI-2 | 3.4 (2.6-4.2) | 2.3 (2.0-2.9) | **<0.001** |
| Insulinogenic index/HOMA-IR | 0.4 (0.3-0.7) | 0.3 (0.2-0.5) | **0.003** |
| Fasting Total cholesterol (mmol/L) | 5.9 (5.3-7.0) | 6.3 (5.7-7.1) | 0.164 |
| Fasting HDL (mmol/L) | 1.9 (1.6-2.4) | 1.9 (1.6-2.2) | 0.692 |
| Fasting LDL (mmol/L) | 3.2 (2.6-4.1) | 3.4 (2.9-4.2) | 0.255 |
| Fasting TG (mmol/L) | 1.6 (1.3-2.0) | 1.8 (1.4-2.3) | **0.027** |
| IPAQ low | 6.2 (2) | 16.5 (250) | 0.148 |
| **Delivery** | | | |
| Total Weight gain (first visit till delivery) (Kg) | 10.9 ± 3.4 | 12.2 ± 5.1 | **0.045** |
| % Excessive weight gain | 4.0 (1) | 28.8 (382) | **0.003** |
| % Inadequate weight gain | 68.0 (17) | 31.1 (413) | **<0.001** |
| Gestational age (weeks) | 39.5 ± 1.3 | 39.3 ± 1.6 | 0.589 |
| % Preeclampsia | 0.0 (0) | 1.8 (29) | 1.000 |
| % Gestational hypertension | 2.9 (1) | 4.3 (67) | 1.000 |
| % Preterm delivery | 5.7 (2) | 5.4 (84) | 0.712 |
| % Induction labor | 31.4 (11) | 25.8 (405) | 0.440 |
| % Cesarean sections (total) | 20.0 (7) | 20.2 (317) | 1.000 |
| Weight baby (g) | 3376.4 ± 340.4 | 3398.4 ±513.1 | 0.481 |
| % Weight baby <2.5 kg | 0.0 (0) | 4.2 (66) | 0.398 |
| % Macrosomia (>4Kg) | 5.7 (2) | 9.5 (149) | 0.767 |
| % LGA | 8.6 (3) | 13.0 (203) | 0.612 |
| % SGA | 5.7 (2) | 5.0 (79) | 0.697 |
| % Neonatal hypoglycemia <2.2 mmol/L | 6.2 (1) | 4.0 (40) | 0.483 |
| % NICU admission | 0.0 (0) | 9.8 (153) | **0.043** |

OGTT: oral glucose tolerance test; GCT: glucose challenge test; GDM: gestational diabetes mellitus; BMI: Body Mass Index; HOMA-IR: Homeostatic Model Assessment for Insulin Resistance; HOMA-B: : Homeostatic Model Assessment for B-cell secretion; ISSI-2: Insulin Secretion-Sensitivity Index-2 HDL: high-density lipoprotein; LDL: low-density-lipoprotein; TG: triglycerides; IPAQ: International Physical Activity Questionnaire; LGA: large-for-gestational age infant; SGA: small-for-gestational age infant; NICU: neonatal intensive care unit; IFG: impaired fasting glycemia; IGT: impaired glucose tolerance; Overweight: BMI ≥25-29.9 Kg/m²; Obesity: BMI ≥30 Kg/m. Categorical variables are presented as frequencies %(n); continuous variables are presented as mean ±SD if normally distributed and as median ± IQR if not normally distributed; Differences are considered significant at p-value<0.05.

**Appendix II: Comparison of characteristics and pregnancy outcomes between women stratified in four groups according to the lowest glycemic values fasting, 1-hour or 2-hour during the OGTT**

|  | Group 1 (<3.9 mmol/L)  N= 172  (10.7 %) | Group 2 (3.9-4.2 mmol/L)  N=441 (27.4%) | Group 3 (4.25-4.4 mmol/L)  N=517 (32.1%) | Group 4 (>4.4 mmol/L)  N=482 (29.9%) | Pairwise comparisons (p-value) | | | | | |
| --- | --- | --- | --- | --- | --- | --- | --- | --- | --- | --- |
|  |  |  |  |  | 1vs2 | 1vs3 | 1vs4 | 2vs3 | 2vs4 | 3vs4 |
| **General** | | | | | | | | | | |
| Age (years) | 29.9 ± 3.9 | 30.4 ± 3.8 | 30.6 ±3.8 | 31.1 ± 4.1 | 0.080 | 0.045 | **<0.001** | 0.746 | **0.015** | **0.026** |
| % Ethnic minorities | 6.4 (11) | 6.6 (29) | 7.8 (40) | 10.9 (52) | 0.949 | 0.556 | 0.091 | 0.468 | **0.021** | 0.093 |
| % multiparity | 45.3 (78) | 44.0 (194) | 44.7 (231) | 50.8 (245) | 0.761 | 0.879 | 0.217 | 0.830 | **0.038** | 0.052 |
| % paid job | 94.1 (161) | 92.9 (490) | 92.6 (477) | 89.0 (427) | 0.595 | 0.497 | **0.049** | 0.843 | **0.036** | **0.045** |
| % living without partner | 17.1 (29) | 15.3 (67) | 15.6 (80) | 20.2 (97) | 0.585 | 0.651 | 0.378 | 0.887 | 0.052 | 0.06 |
| % smoking before pregnancy | 25.3 (43) | 25.2 (111) | 31.7 (163) | 29.2 (140) | 0.975 | 0.114 | 0.335 | 0.026 | 0.174 | 0.384 |
| % smoking during pregnancy | 2.9 (5) | 2.9 (13) | 2.3 (12) | 4.6 (22) | 0.987 | 0.662 | 0.350 | 0.547 | 0.195 | **0.050** |
| % alcohol before pregnancy | 69.6 (119) | 67.3 (296) | 71.9 (371) | 66.3 (317) | 0.582 | 0.563 | 0.434 | 0.121 | 0.759 | 0.057 |
| % alcohol during pregnancy | 8.2 (14) | 5.7 (25) | 7.4 (38) | 6.3 (30) | 0.252 | 0.729 | 0.390 | 0.288 | 0.704 | 0.486 |
| % First degree family history of diabetes | 8.8 (14) | 10.2 (43) | 9.4 (48) | 10.4 (48) | 0.611 | 0.701 | 0.571 | 0.850 | 0.940 | 0.786 |
| % History of GDM* | 5.1 (4) | 4.0 (8) | 5.1 (12) | 6.6 (16) | 0.671 | 0.982 | 0.649 | 0.588 | 0.231 | 0.484 |
| % History of macrosomia >4Kg* | 4.1 (7) | 3.2 (14) | 6.6 (34) | 7.9 (38) | 0.845 | 0.426 | 0.172 | 0.051 | **0.003** | 0.147 |
| **6-14 weeks** | | | | | | | | | | |
| BMI (Kg/m²) | 22.7 ± 3.9 | 23.6 ± 3.9 | 24.2 ± 4.2 | 25.9 ± 5.0 | **0.003** | **<0.001** | **<0.001** | **0.011** | **<0.001** | **<0.001** |
| % Underweight  % Overweight  % Obesity | 4.1 (7)  19.3 (33)  5.8 (10) | 3.6 (16)  29.4 (129)  7.3 (32) | 2.3 (11)  32.4 (167)  9.9 (51) | 2.1 (10)  51.7 (246)  17.6 (84) | 0.794  **0.011**  0.528 | 0.164  **0.001**  0.108 | 0.162  **<0.001**  **<0.001** | 0.160  0.321  0.156 | 0.160  **<0.001**  **<0.001** | 0.973  **<0.001**  **<0.001** |
| % Waist ≥80cm | 62.1 (100) | 70.0 (297) | 75.0 (373) | 81.1 (374) | 0.066 | **0.002** | **<0.001** | 0.089 | **<0.001** | **0.023** |
| Systolic blood pressure (mmHg) | 113.6 ± 9.9 | 113.9 ± 10.2 | 114.9 ± 10.3 | 116.0 ± 10.8 | 0.784 | 0.135 | **0.008** | 0.115 | **0.001** | 0.103 |
| Diastolic blood pressure (mmHg) | 68.2 ± 7.1 | 70.0 ± 7.7 | 70.4 ± 8.1 | 71.3 ± 8.6 | 0.020 | 0.003 | **<0.001** | 0.465 | **0.020** | 0.087 |
| Fasting glycemia (mmol/L) | 4.3 (4.1-4.4) | 4.4 (4.2-4.6) | 4.6 (4.4-4.7) | 4.7 (4.6-4.8) | **<0.001** | **<0.001** | **<0.001** | **<0.001** | **<0.001** | **<0.001** |
| Fasting glycemia <3.9 mmol/L | 10.5 (18) | 1.4 (6) | 1.2 (6) | 0 (0) | **<0.001** | **<0.001** | **<0.001** | 0.781 | **0.010** | **0.018** |
| HOMA-IR | 1.0 (0.7-1.4) | 1.2 (0.9-1.7) | 1.3 (0.9-1.8) | 1.6 (1.2-2.2) | **<0.001** | **<0.001** | **<0.001** | **0.003** | **<0.001** | **<0.001** |
| HOMA-B | 133.7 (92.6-204.3) | 141.4 (105.8-195.0) | 127.4 (92.2-176.9) | 124.8 (93.6-178.4) | 0.409 | 0.158 | 0.243 | **<0.001** | **0.002** | 0.772 |
| HbA1c (mmol/mol and %) | 30 (29-32)  4.9 (4.7-5.1) | 30 (29-32)  4.9 (4.8-5.1) | 31 (29-32)  5.0 (4.8-5.1) | 31 (30-33)  5.0 (4.9-5.2) | 0.195 | **0.001** | **<0.001** | **0.009** | **<0.001** | **<0.001** |
| Fasting Total cholesterol (mmol/L) | 4.6 (4.1-5.2) | 4.6 (4.2-5.1) | 4.6 (4.1-5.2) | 4.7 (4.2-5.3) | 0.431 | 0.372 | 0.042 | 0.846 | 0.051 | 0.099 |
| Fasting HDL (mmol/L) | 1.8 (1.5-2.1) | 1.8 (1.6-2.0) | 1.8 (1.6-2.0) | 1.7 (1.5-1.9) | 0.166 | 0.141 | **<0.001** | 0.839 | **<0.001** | **<0.001** |
| Fasting LDL (mmol/L) | 2.3 (1.9-2.7) | 2.4 (2.0-2.8) | 2.3 (1.9-2.9) | 2.5 (2.0-2.9) | 0.052 | 0.090 | **<0.001** | 0.846 | **0.006** | **0.004** |
| Fasting TG (mmol/L) | 1.0 (0.8-1.1) | 1.0 (0.8-1.2) | 0.9 (0.8-1.2) | 1.0 (0.8-1.3) | 0.918 | 0.705 | **0.040** | 0.518 | **0.013** | **0.002** |
| Total Score lifestyle  Physical activity  Diet | 1.0 (0.0-2.0)  2.0 (0.0-5.0) | 1.0 (0.0-2.0)  2.0 (0.0-4.0) | 1.0 (0.0-2.0)  2.0 (0.0-4.0) | 1.0 (0.0-2.0)  2.0 (-1.0-4.0) | 0.939  0.526 | 0.791  0.180 | 0.062  0.176 | 0.655  0.323 | **0.011**  0.340 | **0.023**  0.965 |
| **24-28 weeks** | | | | | | | | | | |
| Weight gain (first visit till OGTT) (Kg) | 6.8 ± 2.7 | 6.8 ± 3.8 | 7.4 ± 3.0 | 7.1 ± 3.4 | 0.184 | **0.013** | 0.315 | 0.153 | 0.722 | 0.069 |
| BMI (Kg/m²) | 25.2 ± 3.8 | 26.0 ± 3.9 | 26.9 ± 4.2 | 28.5 ± 4.8 | **0.004** | **<0.001** | **<0.001** | **<0.001** | **<0.001** | **<0.001** |
| Systolic blood pressure (mmHg) | 111.9 ± 10.5 | 112.5 ± 9.6 | 112.9 ± 9.8 | 114.4 ± 10.4 | 0.534 | 0.240 | **0.006** | 0.431 | **0.003** | **0.024** |
| Diastolic blood pressure (mmHg) | 65.9 ± 7.9 | 66.2 ± 7.9 | 66.5 ± 7.8 | 68.7 ± 7.8 | 0.630 | 0.342 | **<0.001** | 0.552 | **<0.001** | **<0.001** |
| Glucose non-fasting 0 min on GCT (mmol/L) | 4.5 ± 0.8 | 4.7 ± 0.8 | 4.9 ± 0.9 | 5.1 ± 0.9 | **0.001** | **<0.001** | **<0.001** | **0.001** | **<0.001** | **<0.001** |
| % Glucose <3.9 mmol/L non-fasting 0min on GCT | 19.6 (33) | 11.3 (48) | 6.2 (31) | 2.7 (13) | **0.008** | **<0.001** | **<0.001** | **0.005** | **<0.001** | **0.010** |
| % Glucose <3.5 mmol/L non-fasting 0min on GCT | 7.7 (13) | 3.1 (13) | 1.6 (8) | 0.6 (3) | **0.012** | **<0.001** | **<0.001** | 0.134 | **0.006** | 0.158 |
| Glucose 60 min on GCT (mmol/L) | 6.2 ± 1.4 | 6.4 ± 1.4 | 6.5 ± 1.4 | 6.6 ± 1.4 | 0.059 | **0.004** | **<0.001** | 0.167 | **0.025** | 0.401 |
| % Glucose <3.9 mmol/L 60min on GCT | 2.3 (4) | 3.1 (13) | 1.6 (8) | 0.8 (4) | 0.689 | 0.995 | 0.128 | 0.585 | 0.186 | 0.060 |
| % Glucose <3.5 mmol/L 60min on GCT | 0.6 (1) | 0.2 (1) | 1.4 (7) | 0.4 (2) | 0.492 | 0.409 | 0.785 | 0.056 | 0.617 | 0.116 |
| Fasting glycemia (mmol/L) | 3.8 (3.7-3.9) | 4.0 (4.0-4.2) | 4.3 (4.3-4.4) | 4.7 (4.6-4.8) | **<0.001** | **<0.001** | **<0.001** | **<0.001** | **<0.001** | **<0.001** |
| 30 min glucose OGTT (mmol/L) | 6.3 (5.6-7.1) | 6.7 (6.0-7.4) | 6.9 (6.3-7.6) | 7.2 (6.7-7.9) | **<0.001** | **<0.001** | **<0.001** | **0.008** | **<0.001** | **<0.001** |
| 1-hour glucose OGTT (mmol/L) | 6.0 (3.8-6.0) | 6.5 (4.8-7.6) | 6.8 (6.0-7.8) | 7.3 (6.4-8.3) | **<0.001** | **<0.001** | **<0.001** | **0.013** | **<0.001** | **<0.001** |
| 2-hour glucose OGTT (mmol/L) | 4.9 (3.8-6.0) | 5.8 (4.9-6.7) | 6.0 (5.3-6.9) | 6.3 (5.5-7.1) | **<0.001** | **<0.001** | **<0.001** | **<0.001** | **<0.001** | **<0.001** |
| HbA1c  (mmol/mol and %) | 29 (28-30)  4.8 (4.6-4.9) | 29 (28-31)  4.8 (4.6-5.0) | 30 (29-32)  4.9 (4.8-5.1) | 31 (29-32)  5.0 (4.8-5.1) | 0.057 | **<0.001** | **<0.001** | **<0.001** | **<0.001** | **<0.001** |
| Matsuda insulin sensitivity | 5.9 (4.1-7.6) | 4.6 (3.6-6.0) | 4.0 (3.0-5.5) | 3.1 (2.4-4.2) | **<0.001** | **<0.001** | **<0.001** | **<0.001** | **<0.001** | **<0.001** |
| HOMA-IR | 1.2 (0.8-1.7) | 1.4 (1.1-1.9) | 1.7 (1.3-2.3) | 2.4 (1.7-3.1) | **<0.001** | **<0.001** | **<0.001** | **<0.001** | **<0.001** | **<0.001** |
| HOMA-B | 409.5 (237.2-619.5) | 271.7 (201.5-373.8) | 210.3 (156.6-284.7) | 189.9 (141.1-260.4) | **<0.001** | **<0.001** | **<0.001** | **<0.001** | **<0.001** | **<0.001** |
| ISSI-2 | 3.3 (2.5-4.0) | 2.7 (2.3-3.2) | 2.3 (2.0-2.8) | 1.9 (1.7-2.3) | **<0.001** | **<0.001** | **<0.001** | **<0.001** | **<0.001** | **<0.001** |
| Insulinogenic index/HOMA-IR | 0.5 (0.3-0.8) | 0.4 (0.3-0.6) | 0.3 (0.2-0.4) | 0.2 (0.2-0.3) | **<0.001** | **<0.001** | **<0.001** | **<0.001** | **<0.001** | **<0.001** |
| Fasting Total cholesterol (mmol/L) | 6.2 (5.5-7.2) | 6.3 (5.6-7.0) | 6.3 (5.7-7.0) | 6.3 (5.7-7.1) | 0.837 | 0.759 | 0.852 | 0.913 | 0.962 | 0.969 |
| Fasting HDL (mmol/L) | 2.0 (1.6-2.4) | 1.9 (1.7-2.2) | 2.0 (1.7-2.2) | 1.9 (1.6-2.1) | 0.422 | 0.319 | **<0.001** | 0.939 | **<0.001** | **<0.001** |
| Fasting LDL (mmol/L) | 3.4 (2.8-4.2) | 3.4 (2.9-4.1) | 3.4 (2.9-4.1) | 3.5 (2.9-4.2) | 0.912 | 1.000 | 0.689 | 0.907 | 0.639 | 0.566 |
| Fasting TG (mmol/L) | 1.7 (1.4-2.2) | 1.7 (1.4-2.2) | 1.8 (1.4-2.3) | 1.9 (1.5-2.4) | 0.807 | 0.598 | **0.002** | 0.761 | **<0.001** | **<0.001** |
| Total score lifestyle  Physical activity  Diet | 1.5 (0.0-2.0)  2.0 (0.0-5.0) | 1.0 (0.0-2.0)  2.0 (0.0-4.0) | 1.0 (0.0-2.0)  2.0 (0.0-4.0) | 1.0 (0.0-2.0)  2.0 (-1.0-4.0) | 0.880  0.273 | 0.671  0.483 | 0.226  0.052 | 0.611  0.577 | 0.138  0.221 | 0.328  0.061 |
| % IPAQ low | 13.9 (23) | 15.1 (64) | 17.4 (86) | 17.1 (79) | 0.711 | 0.290 | 0.336 | 0.343 | 0.417 | 0.899 |
| **Delivery** | | | | | | | | | | |
| Total Weight gain (first visit till delivery) (Kg) | 11.3 ± 4.1 | 12.4 ± 5.2 | 12.5 ± 4.5 | 12.0 ± 5.6 | **0.002** | **0.005** | **0.062** | 0.884 | 0.164 | 0.221 |
| % excessive weight gain | 15.3 (20) | 23.2 (86) | 31.4 (136) | 33.8 (141) | 0.056 | **<0.001** | **<0.001** | **0.009** | **0.001** | 0.455 |
| % inadequate weight gain | 51.1 (67) | 31.5 (117) | 28.4 (123) | 29.5 (123) | **<0.001** | **<0.001** | **<0.001** | 0.334 | 0.535 | 0.726 |
| Gestational age (weeks) | 39.0 ± 1.7 | 39.2 ± 1.8 | 39.4 ± 1.6 | 39.4 ± 1.5 | 0.051 | **0.007** | **0.026** | 0.330 | 0.687 | 0.614 |
| % Preeclampsia | 1.7 (3) | 2.3 (10) | 1.0 (5) | 2.3 (11) | 0.690 | 0.408 | 0.682 | 0.107 | 0.992 | 0.100 |
| % Gestational hypertension | 2.3 (4) | 3.6 (16) | 2.9 (15) | 6.8 (33) | 0.419 | 0.692 | **0.029** | 0.529 | **0.030** | **0.004** |
| % Preterm delivery | 7.0 (12) | 6.6 (29) | 4.9 (25) | 4.2 (20) | 0.880 | 0.294 | 0.143 | 0.245 | 0.097 | 0.588 |
| % Induction labor | 16.3 (28) | 23.7 (104) | 24.9 (128) | 32.4 (156) | **0.044** | **0.020** | **<0.001** | 0.678 | **0.003** | **0.009** |
| % Caesarean sections (total) | 19.2 (33) | 16.9 (74) | 19.5 (100) | 24.3 (117) | 0.091 | 0.510 | 0.127 | 0.169 | 0.810 | 0.244 |
| % Emergency CS (during labor) | 7.6 (13) | 7.8 (34) | 9.6 (49) | 13.3 (64) | 0.932 | 0.426 | **0.045** | 0.325 | **0.007** | 0.064 |
| Weight baby (g) | 3286.3 ± 534.4 | 3332.6 ± 493.5 | 3425.0 ± 498.2 | 3468.6 ± 515.6 | 0.322 | **0.002** | **<0.001** | **0.004** | **<0.001** | 0.366 |
| % Weight baby <2.5 kg  Of which:  % SGA  % Preterm delivery  % Intra-uterine growth restriction | 5.8 (10)  60.0 (6)  40.0 (4)  0 (0) | 5.0 (22)  31.8 (7)  59.1 (13)  0 (0) | 4.9 (25)  40.0 (10)  64.0 (16)  0 (0) | 1.9 (9)  33.3 (3)  55.6 (5)  0 (0) | 0.693  0.132  0.316  - | 0.628  0.283  0.195  - | **0.009**  0.245  0.498  - | 0.916  0.560  0.730  - | **0.009**  0.935  0.856  - | **0.010**  0.724  0.655  - |
| % Macrosomia (>4Kg) | 6.4 (11) | 6.9 (30) | 9.6 (49) | 12.7 (61) | 0.835 | 0.201 | **0.023** | 0.130 | **0.003** | 0.118 |
| % Weight baby ≥4.5Kg | 0.6 (1) | 0.5 (2) | 1.4 (7) | 2.3 (11) | 0.843 | 0.408 | 0.151 | 0.149 | **0.019** | 0.272 |
| % LGA | 9.9 (17) | 8.4 (37) | 13.7 (70) | 17.1 (82) | 0.560 | 0.205 | **0.026** | **0.011** | **<0.001** | 0.136 |
| % SGA | 7.6 (13) | 5.7 (25) | 4.5 (23) | 4.2 (20) | 0.393 | 0.116 | 0.079 | 0.389 | 0.273 | 0.796 |
| %Shoulder dystocia | 0.6 (1) | 2.0 (9) | 0.8 (4) | 0.8 (4) | 0.198 | 0.785 | 0.744 | 0.096 | 0.120 | 0.933 |
| % Neonatal hypoglycemia <2.2 mmol/L | 2.8 (3) | 2.2 (6) | 5.2 (17) | 4.7 (15) | 0.717 | 0.310 | 0.404 | 0.056 | 0.099 | 0.771 |
| % NICU admission | 8.2 (14) | 8.0 (35) | 11.1 (57) | 9.8 (47) | 0.927 | 0.287 | 0.535 | 0.107 | 0.330 | 0.519 |

OGTT: oral glucose tolerance test; GCT: glucose challenge test; GDM: gestational diabetes mellitus; BMI: Body Mass Index; HOMA-IR: Homeostatic Model Assessment for Insulin Resistance; HOMA-B: : Homeostatic Model Assessment for B-cell secretion; ISSI-2: Insulin Secretion-Sensitivity Index-2 HDL: high-density lipoprotein; LDL: low-density-lipoprotein; TG: triglycerides; IPAQ: International Physical Activity Questionnaire; LGA: large-for-gestational age infant; SGA: small-for-gestational age infant; NICU: neonatal intensive care unit; IFG: impaired fasting glycemia; IGT: impaired glucose tolerance; Overweight: BMI ≥25-29.9 Kg/m²; Obesity: BMI ≥30 Kg/m. Categorical variables are presented as frequencies %(n); continuous variables are presented as mean ±SD if normally distributed and as median ± IQR if not normally distributed; Differences are considered significant at p-value<0.05. *A history of GDM and a history of a macrosomic baby were calculated on the number of women with a previous pregnancy

**Appendix III: Cut-off fasting glycemia for low birth weight (<2.5kg): sensitivity and specificity with optimal cut-off based on Youden index**

| **Sensitivity** | **Specificity** | **Cut-off** | **Youden-index** | **Max. Youden-index** |
| --- | --- | --- | --- | --- |
| 0.000 | 0.999 | 48 | 0 | . |
| 0.000 | 0.998 | 50 | 0 | . |
| 0.000 | 0.997 | 57 | 0 | . |
| 0.000 | 0.997 | 58 | 0 | . |
| 0.000 | 0.996 | 60 | 0 | . |
| 0.000 | 0.995 | 61 | 0 | . |
| 0.000 | 0.994 | 62 | 0 | . |
| 0.000 | 0.991 | 63 | 0 | . |
| 0.030 | 0.986 | 64 | .02 | . |
| 0.030 | 0.981 | 65 | .01 | . |
| 0.045 | 0.973 | 66 | .02 | . |
| 0.076 | 0.965 | 67 | .04 | . |
| 0.091 | 0.947 | 68 | .04 | . |
| 0.136 | 0.926 | 69 | .06 | . |
| 0.182 | 0.896 | 70 | .08 | . |
| 0.212 | 0.862 | 71 | .07 | . |
| 0.288 | 0.817 | 72 | 0.1 | . |
| 0.364 | 0.777 | 73 | .14 | . |
| 0.424 | 0.723 | 74 | .15 | . |
| 0.439 | 0.655 | 75 | .09 | . |
| 0.561 | 0.594 | 76 | .16 | . |
| 0.636 | 0.531 | 77 | .17 | . |
| 0.682 | 0.455 | 78 | .14 | . |
| 0.727 | 0.392 | 79 | .12 | . |
| **0.848** | **0.329** | **80** | **.18** | **Maximum Youden index** |
| 0.848 | 0.257 | 81 | .11 | . |
| 0.864 | 0.212 | 82 | .08 | . |
| 0.909 | 0.174 | 83 | .08 | . |
| 0.939 | 0.137 | 84 | .08 | . |
| 0.955 | 0.105 | 85 | .06 | . |
| 0.955 | 0.076 | 86 | 0 | . |
| 0.955 | 0.058 | 87 | 0 | . |
| 0.955 | 0.040 | 88 | 0 | . |
| 0.985 | 0.022 | 89 | 0 | . |
| 1.000 | 0.010 | 90 | 0 | . |
| 1.000 | 0.000 | 91 | 0 | . |
